# *Heracleum moellendorffii* root extracts exert immunostimulatory activity through TLR2/4‐dependent MAPK activation in mouse macrophages, RAW264.7 cells

**DOI:** 10.1002/fsn3.2020

**Published:** 2020-11-16

**Authors:** Ho Jun Son, Hyun Ji Eo, Gwang Hun Park, Jin Boo Jeong

**Affiliations:** ^1^ Forest Medicinal Resources Research Center National Institute of Forest Science Yeongju Korea; ^2^ Department of Medicinal Plant Resources Andong National University Andong Korea

**Keywords:** *H. moellendorffii* Root, immune enhancement, immunomodulators, macrophages, wild simulated ginseng

## Abstract

*Heracleum moellendorffii* (*H. moellendorffii*) is a family of Umbelliferae and has long been used for food and medicinal purposes. However, the immune‐enhancing activity of *H. moellendorffii* has not been studied. Thus, we evaluated in vitro immune‐enhancing activity of *H. moellendorffii* through macrophage activation using RAW264.7 cells. *Heracleum moellendorffii* Root extracts (HMR) increased the production of immunomodulators such as NO, iNOS, IL‐1β, IL‐6 IL‐12, TNF‐α, and MCP‐1 and activated phagocytosis in RAW264.7 cells. Inhibition of TLR2 and TLR4 reduced the production of immunomodulators induced by HMR. Inhibition of MAPK signaling attenuated the production of immunomodulators induced by HMR, but inhibitions of NF‐κB or PI3K/AKT signaling did not affect HMR‐mediated production of immunomodulators. HMR activated MAPK signaling pathway, and activation of MAPK signaling pathways by HMR was reversed by TLR2 and TLR4 inhibition. Based on the results of this study, HMR is thought to activate macrophages through the production of immunomodulators and phagocytosis activation through TLR2/4‐dependent MAPK signaling pathway. Therefore, it is thought that HMR has the potential to be used as an agent for enhancing immunity.

## Graphical abstract

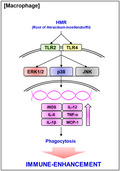

## 1 INTRODUCTION

It is known that macrophages among immune cells contribute to maintaining resistance to external pathogens and homeostasis of the human body through innate and adaptive immune responses (Daliri, Choi, et al., 2019; Hirayama et al., 2018). Activated macrophage secrete various immunostimulatory factors such as nitric oxide (NO), inducible nitric oxide synthase (iNOS), interleukin‐1β (IL‐1β), interleukin‐6 (IL‐6), interleukin‐12 (IL‐12), tumor necrosis factor‐α (TNF‐α), and monocyte chemoattractant protein‐1 (MCP‐1), which are known to perform innate immune responses by destroying external pathogens that have penetrated into the human body (Hirayama et al., 2018). In addition, these immunostimulatory factors induce macrophage phagocytosis to engulf and remove external pathogens (Martinez & Gordon, 2015). Macrophages have the ability to present antigens to T cells and also function as effectors of cell‐mediated immunity (Daliri, Kim, et al., 2019; Hirayama et al., 2018). In addition, various immunostimulatory factors produced by macrophages have been reported to activate T cells and B cells (Bordet et al., 2019; Craxton et al., 2003; Guerriero, 2019; Lapaque et al., 2009). These reports show that macrophages can contribute to the adaptive immune response as well as the innate immune response. Therefore, it has been recognized that activation of macrophages related to innate immunity and adaptive immunity helps strengthen the body's immune system.

Numerous studies have reported that eating wild vegetables can reduce the risk of various diseases such as cancer, atherosclerosis, cardiovascular disease, inflammation, and diabetes (Xie et al., 2015). Thus, wild vegetables have been regarded as important plant resources for human health in recent decades (Afolayan & Jimoh, 2009; Flyman & Afolayan, 2007). Moreover, may natural products have been used for the development of immunotherapy in the treatment of human diseases (Chen & Yu, 2016). *Heracleum moellendorffii* (*H. moellendorffii*) is a family of Umbelliferae and has long been used for food and medicinal purposes (Alam et al., 2016). The young leaves of *H. moellendorffii*, which have various pharmacological activities, including detoxification and antioxidant activity, have been used as wild vegetables. And the roots of *H. moellendorffii* have traditionally been used to treat diseases related to chronic inflammation among various disease of human (Bang et al., 2009; Kim et al., 2019; Park et al., 2010). Although there have been no studies related to the immune‐enhancing activity of *H. moellendorffii*, several plants in the genus *Heracleum* such as *H. persicum* and *H. maximum* have been reported to enhance the body's immunity (Naeini et al., 2013; Webster et al., 2006). Therefore, in this study, the immune‐enhancing activity through the macrophage activation of *H. moellendorffii* was evaluated, and the mechanism of action was elucidated.

## 2 MATERIALS AND METHODS

### 2.1 Reagents

Dulbecco's Modified Eagle medium (DMEM)/F‐12 1:1 Modified medium was purchased from Lonza. 3‐(4,5‐dimethylthiazol‐2‐yl)‐2,5‐diphenyltetrazolium bromide (MTT), SB203580 (p38 inhibitor), PD98059 (ERK1/2 inhibitor), SP600125 (JNK inhibitor), BAY 11–7082 (IKK inhibitor), TAK‐242 (TLR4 inhibitor), neutral red, and Griess reagent were purchased from Sigma‐Aldrich. C29 (TLR2 inhibitor) was purchased from BioVision. The primary antibodies and secondary antibody were purchased from Cell Signaling. RNeasy Mini Kit was purchased from Qiagen. Verso cDNA Kit was purchased from Thermo Scientific. PCR Master Mix Kit was purchased from Promega.

### 2.2 Sample preparation

The roots and leaves of *H. moellendorffii* (voucher number: FMCHm‐2019–0521–001 ~003) were provided after botanical identification through Forest Medicinal Resources Research Center, National Institute of Forest Science, Yongju, Korea. To prepare the sample, 1 g of *H. moellendorffii* roots and leaves were immersed in 20 ml distilled water, respectively, and then extracted by stirring at 150 rpm for 3 days at 15°C. After 3 days, the extracts were centrifuged at 12,000 rpm for 10 min at 4°C to obtain r a clear supernatant. Then, the recovered supernatant was lyophilized. Freeze‐dried root extracts (HMR) and leave extracts (HML) of *H. moellendorffii* were stored at −80°C until use. HMR or HML was dissolved in sterile distilled water and aliquoted in small portions and stored at −80°C until use.

### 2.3 Cell culture

RAW264.7 cells purchased from American Type Culture Collection were maintained using Dulbecco's Modified Eagle medium (DMEM)/F‐12 1:1 Modified medium (Lonza) containing 10% fetal bovine serum, 100 U/ml penicillin and 100 μg/ml streptomycin at 37°C under a humidified atmosphere of 5% CO_2_.

### 2.4 Measurement of cell viability

Cytotoxicity to RAW264.7 cells was verified using MTT assay. Briefly, RAW264.7 cells (1 × 10^4^ cells/well) were dispensed into 96‐well plates and cultured until 70%–80% confluence. Then, cells were treated with HMR and subsequently cultured for 24 hr. After HMR treatment, cells were added with 50 μl of MTT solution (1 mg/ml) for 4 hr. Then, the cell culture medium was removed and 100 μl of DMSO was treated elute the resulting crystals from RAW264.7 cells. The absorbance was measured at 570 nm using UV/Visible spectrophotometer (Human Cop., Xma‐3000PC).

### 2.5 Measurement of NO

The effect of HMR or HML on NO production in RAW264.7 cells was evaluated by Griess assay. Briefly, RAW264.7 cells (2 × 10^5^ cells/well) were dispensed into 12‐well plates and cultured until 70%–80% confluence. Then, cells were treated with HMR or HML for 24 hr. After HMR treatment, Griess solution was added to the cell culture medium in a 1:1 ratio, and the mixture was incubated at room temperature for 15 min. Then, the absorbance was measured at 540 nm using UV/Visible spectrophotometer (Human Cop., Xma‐3000PC).

### 2.6 Measurement of phagocytic activity

The effect of HMR on macrophage phagocytosis was evaluated by neutral red uptake assay. Briefly, RAW264.7 cells (2 × 10^5^ cells/well) were dispensed into 12‐well plates and cultured until 70%–80% confluence. Then, cells were treated with HMR for 24 hr. After HMR treatment, after washing cells with 1 × PBS, 1 ml of a 0.01% neutral red solution was treated into cells for 2 hr. Then, after washing cells with 1 × PBS, the cells were observed with an optical microscope, and subsequently 1 ml of lysis buffer (ethanol acid: acetic acid = 1:1) was treated to elute the absorbed neutral red. The absorbance was measured at 540 nm using UV/Visible spectrophotometer (Human Cop., Xma‐3000PC).

### 2.7 Western blot analysis

After the sample treatment was completed, the RAW264.7 cells were washed 3 time with cold 1 × PBS, and then the cells were lysed for 30 min at 4°C using RIPA buffer (Boston Bio Products) containing protease inhibitor (Sigma‐Aldrich) and phosphatase inhibitor (Sigma‐Aldrich) to extract cellular proteins from the cells. After 30 min, protein was quantified by BCA protein assay (Thermo Fisher Scientific) using a clear supernatant obtained by centrifugation at 4°C for 10 min. Then, the protein was separated by SDS‐PAGE and subsequently transferred to the PVDF membrane. After blocking the PVDF membrane for 1 hr by stirring at room temperature with a blocking buffer (5% not‐fat dry milk in Tris‐buffered saline containing 0.05% Tween 20 (TBS‐T), the primary antibody was treated and reacted with stirring at 4°C overnight. Then, after washing PVDF membrane with TBS‐T, the secondary antibody was treated and reacted at room temperature for 1 hr. After washing PVDF membrane with TBS‐T, Chemiluminescence was detected with ECL Western blotting substrate (Amersham Biosciences) and visualized using LI‐COR C‐DiGit Blot Scanner (Li‐COR Biosciences). The density of Western blot bands was calculated using the software UN‐SCAN‐IT gel version 5.1 (Silk Scientific Inc.).

### 2.8 Reverse transcription polymerase chain reaction (RT‐PCR)

After the sample treatment was completed, the RAW264.7 cells were washed three time with cold 1 × PBS. Total RNA was extracted from the cells using a RNeasy Mini Kit, and after quantitation of total RNA, cDNA was synthesized using a Verso cDNA Kit (Thermo Scientific) from 1 μg of total RNA. Then, the target genes were amplified using a PCR Master Mix Kit (Promega) and the primers. The sequences of the primers used in this study were as follows: iNOS: forward 5′‐ttgtgcatcgacctaggctggaa‐3′ and reverse 5′‐gacctttcgcattagcatggaagc‐3′, IL‐1β: forward 5′‐ggcaggcagtatcactcatt‐3′ and reverse 5′‐cccaaggccacaggtattt‐3′, IL‐6: forward 5′‐gaggataccactcccaacagacc‐3′ and reverse 5′‐aagtgcatcatcgttgttcataca‐3′, IL‐12: forward 5′‐aaccagacccgcccaagaac‐3′ and reverse 5′‐gatcctgagcttgcacgcaga‐3′, TNF‐α: forward 5ʹ‐tggaactggcagaagaggca‐3ʹ and reverse 5ʹ‐tgctcctccacttggtggtt‐3ʹ, MCP‐1: forward 5′‐ggaaaaatggatccacaccttgc‐3′ and reverse 5′‐tctcttcctccaccaccatgcag‐3′, GAPDH: forward 5′‐ggactgtggtcatgagcccttcca‐3′ and reverse 5′‐actcacggcaaattcaacggcac‐3′. The PCR results were visualized using agarose gel electrophoresis. The density of mRNA bands was calculated using the software UN‐SCAN‐IT gel version 5.1 (Silk Scientific Inc.).

### 2.9 Statistical analysis

All the data are shown as mean ± *SD* (standard deviation). Statistical significance was determined by Student's *t* test. Differences with *p < .05 were considered statistically significant.

## 3 RESULTS AND DISCUSSION

### 3.1 HMR activates mouse macrophage RAW264.7 cells

**FIGURE 1:**
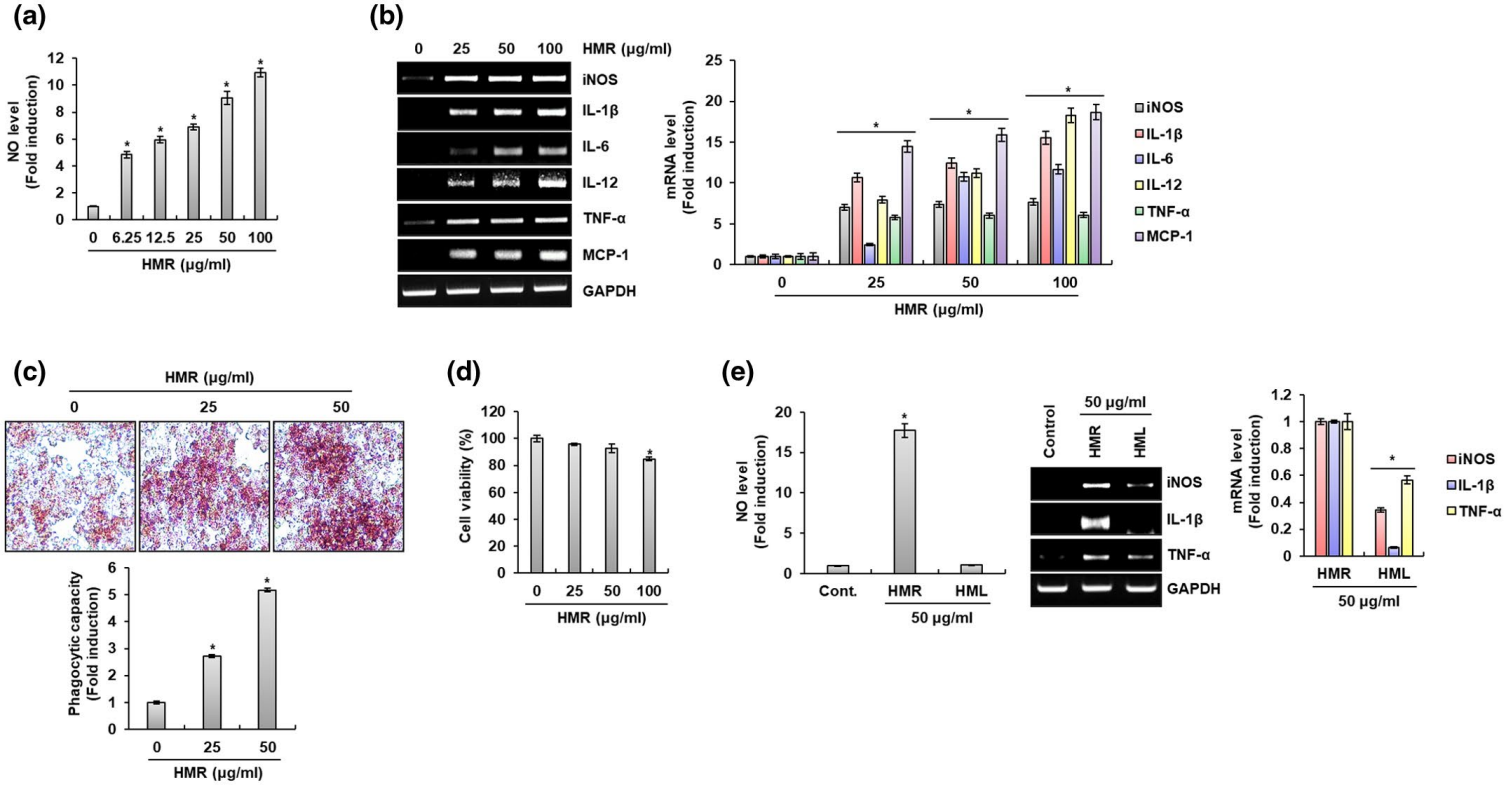
Effect of HMR on macrophage activation. (a) NO level was measured using Griess assay at 24 hr after HMR treatment. (b) mRNA level was investigated using RT‐PCR at 24 hr after HMR treatment (c) Phagocytic capacity was measured using Neutral Red staining at 24 hr after HMR treatment. (d) Cell viability was measured using MTT assay at 24 hr after HMR treatment. (e) NO level or mRNA level was investigated using Griess assay and RT‐PCR at 24 hr after HMR and HML treatment, respectively. *p < .05 compared to the cells without HMR or HML treatment

Macrophages, one of the immune cells with phagocytosis, are known to contribute to the innate and adaptive immune responses to increase the body's immunity (Zhang et al., 2019). Therefore, studies on natural products capable of activating macrophages are being actively conducted to rapidly respond to external pathogens before the adaptive immune response of the human body is activated. The mouse macrophage cell line RAW264.7 cells have been commonly used to evaluate immune‐enhancing activity through macrophage activation in vitro. Thus, we first analyzed the immunomodulatory factors such as NO, iNOS, IL‐1β, IL‐6, IL‐12, TNF‐α, and MCP‐1 secreted by RAW264.7 cells after HMR treatment to evaluate whether HMR activates macrophage. As shown in Figure 1a, HMR promoted NO secretion in macrophages in a dose‐dependent manner. In addition, the expressions of immunomodulators such as iNOS, IL‐1β, IL‐6, IL‐12, TNF‐α, and MCP‐1 were increased by HMR treatment in RAW264.7 cells (Figure 1b). It is well‐known that the innate immune response by macrophages is triggered by the phagocytosis against the external pathogens (Aderem & Underhill, 1999). It is well‐known that these immunomodulators secreted by activated macrophages contribute to the elimination of external pathogens by increasing the phagocytosis of macrophages (Liu et al., 2016). Thus, we analyzed the effect of HMR on the macrophage phagocytosis through Neutral Red staining assay. As shown in Figure 1c, it was observed that the Neutral Red uptake of RAW264.7 cells was increased depending on the treatment concentration of HMR. It is thought that HMR activates the macrophage phagocytosis because increasing of Neutral Red uptake by macrophages indicates the activation of macrophage phagocytosis. However, because it is known that excessive secretion of immunomodulatory factors by macrophages acts as a chronic inflammatory factor or is cytotoxic to macrophages (Taylor et al., 2003), we investigated whether HMR showed cytotoxicity to RAW264.7 cells. As shown in Figure 1d, there was no cytotoxicity against RAW264.7 cells at 25 μg/ml and 50 μg/ml concentrations of HMR. However, at a concentration of 100 μg/ml of HMR, it showed somewhat cytotoxicity to RAW264.7 cells, but the viability of RAW264.7 cells was more than 80%. Based on these results, it is thought that the immunomodulators induced by HMR in RAW264.7 cells may contribute to the enhancement of the immune system of human body. Lastly, we analyzed the production of immunomodulators of the HMR and HML since it is known to that HML also has various pharmacological activities. As shown in Figure 1e, when compared to HMR‐treated cells, NO production was not induced in HML‐treated cells, and the expression of immunomodulators such as NO, iNOS, IL‐1β, and TNF‐α was also weak in HML‐treated cells. In view of this result, it is judged that it may be better to use HMR rather that HML when developing functional agents for enhancing immune system using *H. moellendorffii*.

### 3.2 TLR2/4 contribute to HMR‐mediated production of immunomodulators in RAW264.7 cells

**FIGURE 2:**
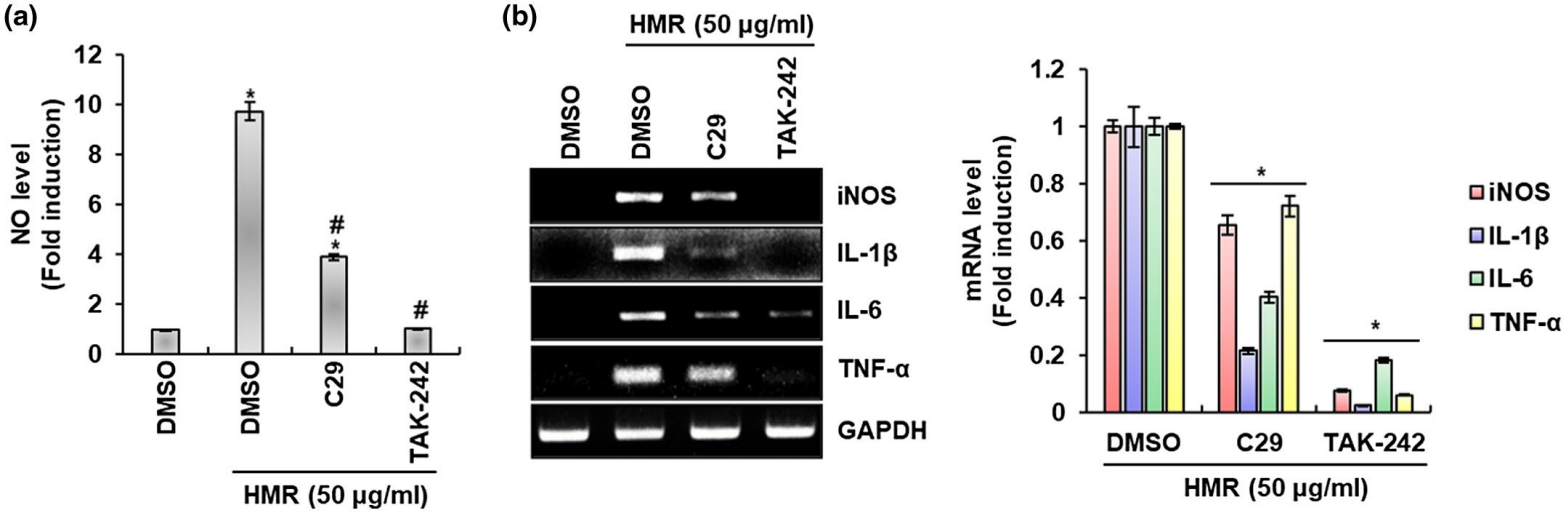
TLR2/4‐dependent macrophage activation by HMR. RAW264.7 cells were pretreated with C29 (100 μM) or TAK‐242 (5 μM) for 2 hr and then co‐treated with HMR (50 μg/ml) for 24 hr. NO level (a) and mRNA level (b) were investigated using Griess assay and RT‐PCR, respectively. *p < .05 compared to the cells without the treatment. ^#^ *p* < .05 compared to the cells with HMR treatment alone

The immune response by macrophages begins when the pattern recognition receptors (PRRs) present in the cell membrane of macrophages recognizes pathogen associated molecular patterns (PAMPs) of external pathogens (Kawai & Akira, 2011; Russo et al., 2017). It is known that Toll‐like receptors (TLRs) contribute to the early recognition of external pathogens by macrophage among PRRs of macrophages (Kawai & Akira, 2011) and TLR signaling has been reported to induce the secretion of various immunomodulators to fight external pathogens (Strieter et al., 2003). Macrophages secretes various immunomodulators through activation of TLR2 and TLR4 among TLRs (Hoogerwerf et al., 2010; Juarez et al., 2010). Thus, we investigated whether TLR2 and TLR4 contribute to the production of immunomodulators such as NO, iNOS, IL‐1β, IL‐6, and TNF‐α. As shown in Figure 2a,b, the inhibition of TLR2 by C29 and TLR4 by TAK‐242 reduced the production of immunomodulators by HMR when compared to the cell treated with HMR alone. In view of this result, both TLR2 and TLR4 may be thought to be receptors that contribute to the production of immunomodulators by HMR in RAW264.7 cells. Indeed, various natural products have been reported to stimulate TLR2 and TLR4 in macrophages to activate the production of immunomodulators (Iwasaki & Medzhitov, 2010; Lee & Hong, 2011; Shen et al., 2017; Yang et al., 2017). In view of the above results, TLR2 and TLR4 may be thought to be important receptors involved in the production of immunomodulators by HMR in macrophages.

### 3.3 MAPK pathways contribute to HMR‐mediated production of immunomodulators in RAW264.7 cells

**FIGURE 3:**
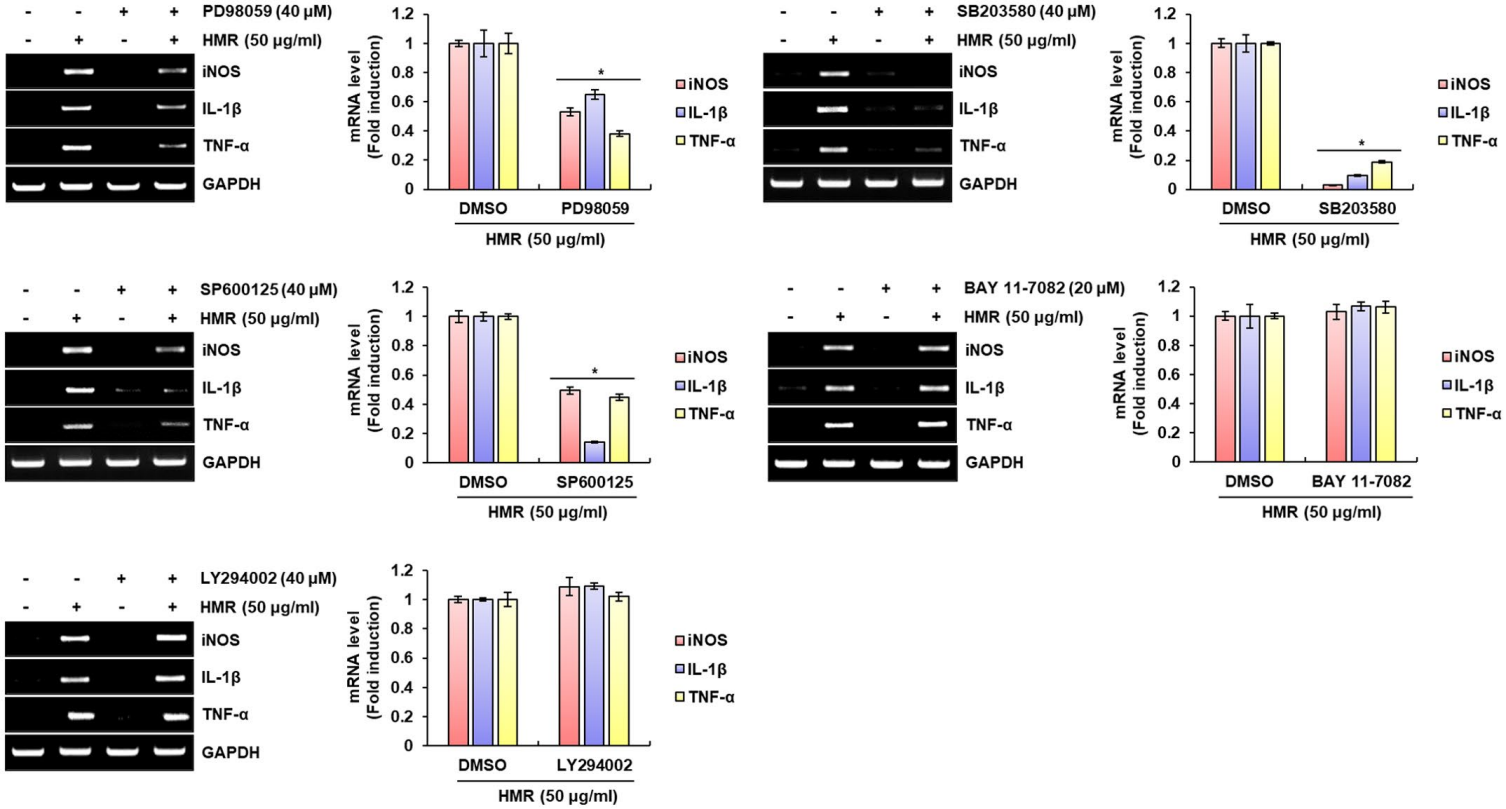
MAPKs‐dependent macrophage activation by HMR. RAW264.7 cells were pretreated with PD98059 (40 μM), SB203580 (40 μM), SP900125 (40 μM), BAY 11–7082 (40 μM), or LY294002 (40 μM) for 2 hr and then co‐treated with HMR (50 μg/ml) for 24 hr. mRNA level was investigated using RT‐PCR. *p < .05 compared to the cells without each inhibitor

In cells involved in innate immunity such as macrophages, mitogen‐activated protein kinases (MAPKs) signaling is activated by PRRs such as TLRs, which is known to be associated with the production of the immunomodulators (Arthur & Ley, 2013). It has also been reported that nuclear factor‐κB (NF‐κB) activated by the stimulation of TLRs is an essential master regulatory signaling pathway that contributes to the production of immunomodulator by macrophages and other innate immune cells against external pathogens (Dorrington & Fraser, 2019; Kawai & Akira, 2007). In addition to MAPKs and NF‐κB signalings, the phosphoinositide 3‐kinase (PI3K)/AKT signaling is also activated by TLRs and induces the production of various immunomodulators (Troutman et al., 2012). Thus, we investigated whether MAPKs, NF‐κB and PI3K/ATK signaling result in the production of immunomodulators such as iNOS, IL‐1β and TNF‐α by HMR in RAW264.7 cells. As shown in Figure 3, the inhibitions of ERK1/2 by PD98059, p38 by SB203580, and JNK by SP600125 reduced the production of iNOS, IL‐1β and TNF‐α by HMR when compared to cells treated with HMR alone. However, when inhibitions of PI3K/AKT signaling by LY294002 and NF‐κB signaling by BAY 11–7082, there was no change in the production of immunomodulators such as iNOS, IL‐1β, and TNF‐α by HMR. Through these results, MAPKs is thought to be an important signaling involved in the production of immunomodulators by HMR in macrophages.

### 3.4 Activation of MAPKs by HMR is dependent on TLR2/TLR4 in RAW264.7 cells

**FIGURE 4:**
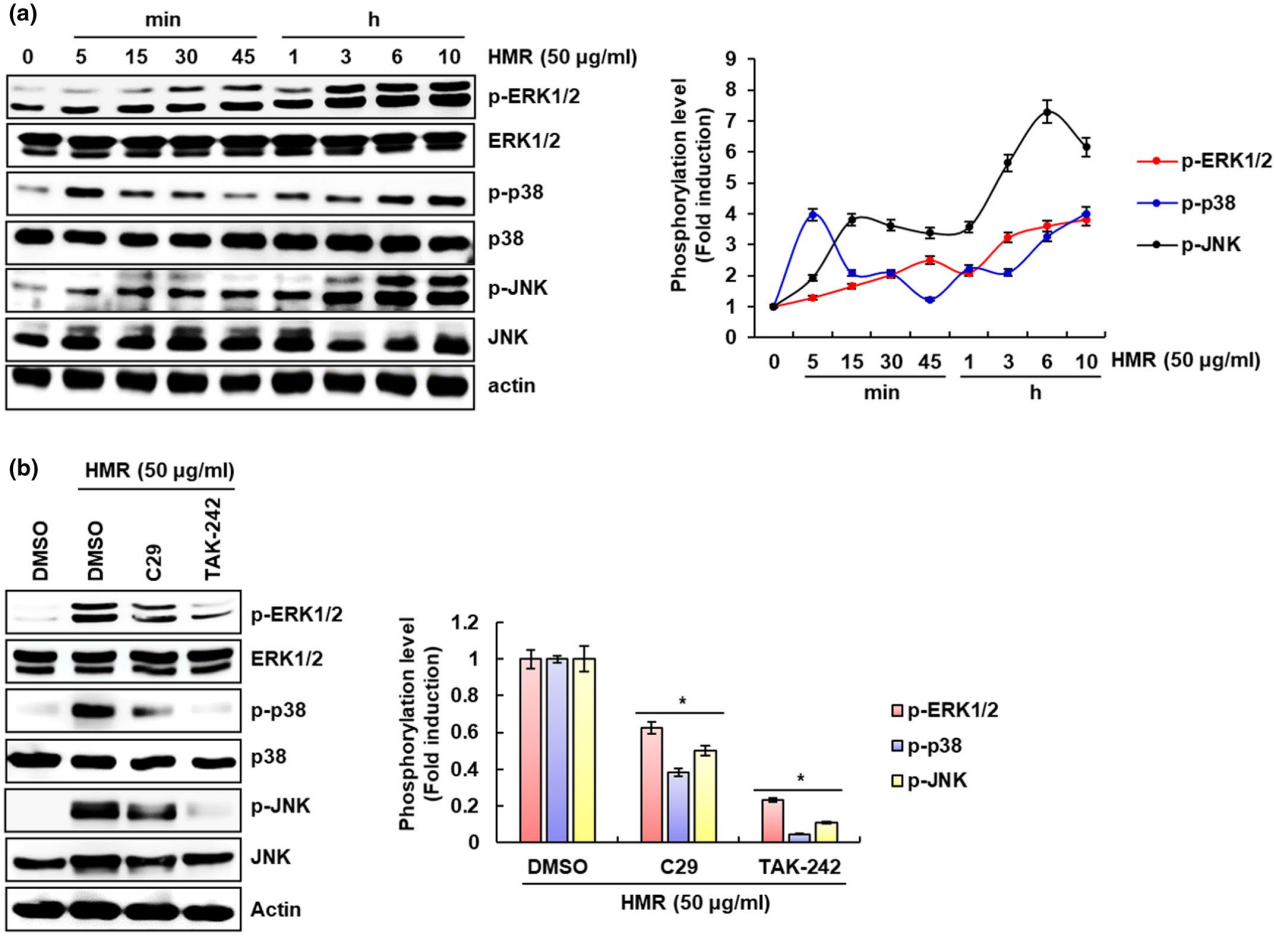
TLR2/4‐dependent activation of MAPK signaling. (a) RAW264.7 cells were treated with HMR (50 μg/ml) for the indicated times. (b) RAW264.7 cells were pretreated with C29 (100 μM) or TAK‐242 (5 μM) for 2 hr and then co‐treated with HMR (50 μg/ml) for 6 hr. Each protein level was investigated using Western blot analysis. Actin was used as a loading control. *p < .05 compared to the cells without HMR or each inhibitor

In Figure 3, we confirmed that HMR depends on MAPKs to induce the production of immunomodulators in RAW264.7 cells. Thus, we investigated whether HMR activates MAPK signaling in RAW264.7 cells. As shown in Figure 4a, HMR induced phosphorylation of ERK1/2, p38 and JNK indicating the MAPK activation. And since TLR2 and TLR4 activate MAPK signaling, we investigated the effect of TLR2 and TLR4 on HMR‐mediated MAPK activation. As a result (Figure 4b), inhibition of TLR2 by C29 and TLR4 by TAK‐242 reduced phosphorylation of ERK1/2, p38, and JNK induced by HMR when compared to cells treated with HMR only. Based on these results, it is thought HMR may induce the activation of MAPK signaling pathway through TLR2 and TLR4.

### 3.5 HMR′s activity on the production of immunomodulators is higher than that of wild simulated ginseng and ginseng

**FIGURE 5:**
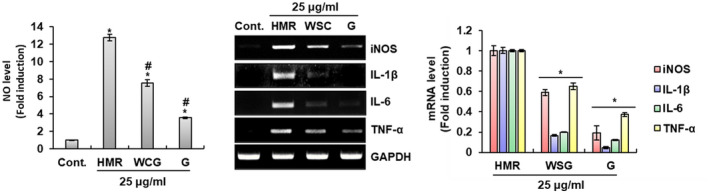
Comparison of macrophage activation of HMR, ginseng and wild simulated ginseng. RAW264.7 cells were treated with HMR (25 μg/ml), G (25 μg/ml) or WSG (25 μg/ml) for 24 hr. NO level and mRNA level were investigated using Griess assay and RT‐PCR, respectively. *p < .05 compared to the cells without the sample treatment

Ginseng (*Panax ginseng* Meyer) has been regarded as one of the representative traditional herbal medicines related to immune enhancement worldwide (Kang & Min, 2012). It has been reported that wild simulated ginseng, a type of ginseng, also increases phagocytosis and promotes the production of various immunomodulators in macrophages (Jung et al., 2019). Thus, we compared the degree of macrophage activation of HMR with ginseng (G) and wild simulated ginseng (WSG) through the production of immunomodulators in RAW264.7 cells. As shown in Figure 5, when compared to cells treated with WSG or G, the production of immunomodulator such as NO, iNOS, IL‐1β, IL‐6, and TNF‐a was highest in HMR‐treated RAW264.7 cells. In view of this result, HMR is a potential herbal resource that can replace ginseng in immunotherapy for the treatment of human diseases.

## 4 CONCLUSION

**FIGURE 6:**
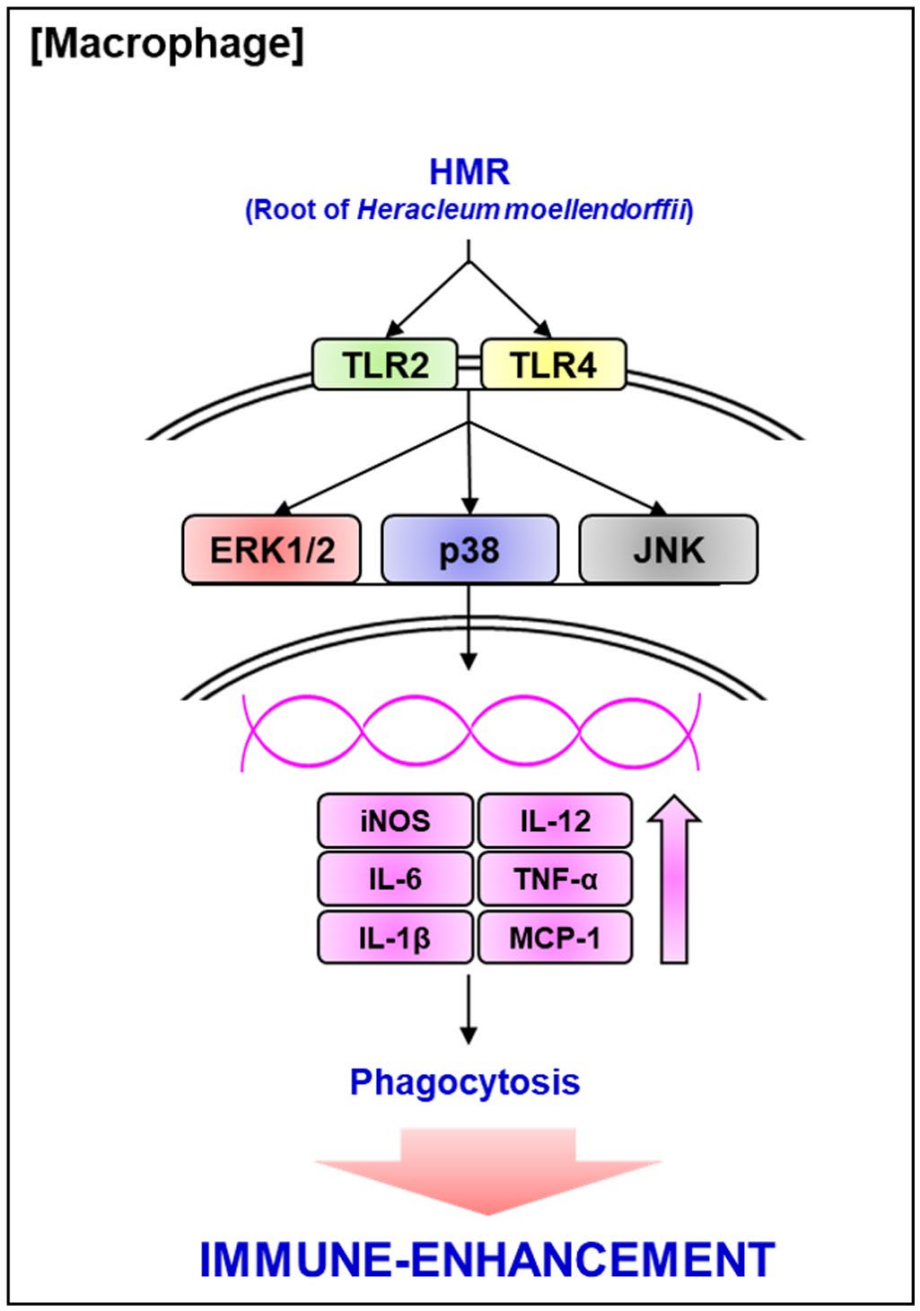
Scheme of the pathway of HMR‐mediated activation of mouse macrophage RAW264.7 cells. HMR increases the production of immunomodulators through the activation of MAPK (ERK1/2, p38 and JNK) pathways via the stimulation of TLR2 and TLR4 in mouse macrophage RAW264.7 cells

In view of the overall data presented in the study, HMR is thought to activate macrophages through the production of immunomodulators and phagocytosis activation through TLR2/4‐dependent MAPK signaling pathways (Figure 6). Because macrophage activation is considered to be one of the targets for enhancing the immune system of the human body, HMR is considered to have the immune‐enhancing activity and can be used as an important natural product for the development of immune‐enhancing agents in the future.

## CONFLICT OF INTEREST

The authors declare no conflict of interest.
